# dsOMOP: bridging OMOP CDM and DataSHIELD for secure federated analysis of standardized clinical data

**DOI:** 10.1093/bioinformatics/btaf286

**Published:** 2025-05-06

**Authors:** David Sarrat-González, Xavier Escribà-Montagut, Jared Houghtaling, Juan R González

**Affiliations:** Bioinformatic Research Group in Epidemiology (BRGE), Barcelona Institute for Global Health (ISGlobal), 08003 Barcelona, Spain; Bioinformatic Research Group in Epidemiology (BRGE), Barcelona Institute for Global Health (ISGlobal), 08003 Barcelona, Spain; Institute for Clinical Research and Health Policy Studies (ICRHPS), Tufts University School of Medicine, Boston, MA 02111, United States; Bioinformatic Research Group in Epidemiology (BRGE), Barcelona Institute for Global Health (ISGlobal), 08003 Barcelona, Spain; CIBER in Epidemiology and Public Health (CIBERESP), Instituto de Salud Carlos III, 28029 Madrid, Spain

## Abstract

**Motivation:**

Collaborative clinical research projects face several challenges related to data sharing. The disparity between data standards and strict privacy regulations become more relevant as the number of involved institutions increases. To address these challenges, the scientific community has progressively adopted common data models like the Observational Medical Outcomes Partnership Common Data Model (OMOP CDM) for multicenter data standardization and implemented federated data analysis platforms like DataSHIELD to perform remote analyses without transferring individual-level data between centers, thus mitigating disclosure risks. However, there is no native implementation that automatically combines both solutions, revealing the need for a tool that enables interoperability between these systems.

**Results:**

We present dsOMOP, a collection of DataSHIELD packages that facilitates automated extraction and transformation of OMOP CDM data into DataSHIELD-compatible datasets, enabling disclosure-controlled federated analyses of standardized clinical data. dsOMOP allows research institutions to provide access to their data for collaborative projects in a format that is interoperable with the project’s available data, thus facilitating the analysis of large-scale, multicenter clinical data. It incorporates OMOP data directly into the DataSHIELD workflow, where all analyses occur entirely in a federated environment subject to rigorous disclosure controls, ensuring that only aggregated, non-disclosive results are ever returned to analysts.

**Availability and implementation:**

The general information page for the dsOMOP environment is available at https://isglobal-brge.github.io/dsOMOP, where the most recent installation instructions and usage guides for all dsOMOP packages and their extensions can be found in the “Packages” section.

The dsOMOP package and its complementary tools are fully available under the MIT license on GitHub: dsOMOP (https://github.com/isglobal-brge/dsOMOP), dsOMOPClient (https://github.com/isglobal-brge/dsOMOPClient), dsOMOPHelper (https://github.com/isglobal-brge/dsOMOPHelper), and dsOMOP.oracle (https://github.com/isglobal-brge/dsOMOP.oracle).

Usage vignettes for the client-side packages are available at the websites of dsOMOPClient (https://isglobal-brge.github.io/dsOMOPClient) and dsOMOPHelper (https://isglobal-brge.github.io/dsOMOPHelper). A permanent archival snapshot of the exact code used in this manuscript is deposited at Figshare: https://doi.org/10.6084/m9.figshare.28607186.

## 1 Introduction

The ability to share and analyze interoperable, large-scale clinical data from multiple centers is fundamental for collaborative clinical research. In this context, the Observational Medical Outcomes Partnership (OMOP) Common Data Model (CDM) has been established as a valuable and widely extended standard for the harmonization of clinical data. The OMOP CDM enables the integration of disparate data from different medical sources, facilitating comparative and integrated analyses (Voss *et al.* 2015), which are essential for multicenter studies.

For instance, in cancer prediction studies, OMOP CDM has enabled the standardization of large volumes of data originating from different clinical centers (Ahmadi *et al.* 2022), which are crucial for the development and optimization of reliable machine learning (ML) models and biomarker discovery. This standardization also improved the reproducibility, reusability, and validity of such studies.

In addition to data standardization, privacy preservation and disclosure control are critical aspects of data sharing in multicenter research. To address these privacy requirements, *DataSHIELD* has emerged as a federated analysis platform that enables analyses of distributed data without the need to physically move data between centers (Gaye *et al.* 2014). This capability of *DataSHIELD* to maintain data governance while conducting meaningful analyses has been widely recognized and applied in various studies, largely because it addresses the ethical issues associated with data sharing and ultimately supports interrogation of individual-level data across multiple sites, thus significantly reducing the risk of patient privacy breaches (Budin-Ljøsne *et al.* 2015). This federated approach aligns with the internationally recognized “Five Safes” framework, emphasizing rigorous control measures to mitigate disclosure risks (Avraam *et al.* 2025). For instance, Wilson *et al.* (2017) explored advanced applications of *DataSHIELD*, including the analysis of sensitive data post-publication, text data processing, and privacy-preserving data visualization, highlighting its versatility and relevance beyond biomedical research.

Despite the progress achieved with OMOP CDM and *DataSHIELD* separately, the lack of an existing solution that combines both tools limits their potential use in collaborative research. Here, we present *dsOMOP*, a solution designed to automatically extract, transform, and load OMOP CDM data into the *DataSHIELD* analytics framework, enabling non-disclosive federated analysis of standardized clinical data.

## 2 Materials and methods

### 2.1 Client–server communication

**Figure 1.**
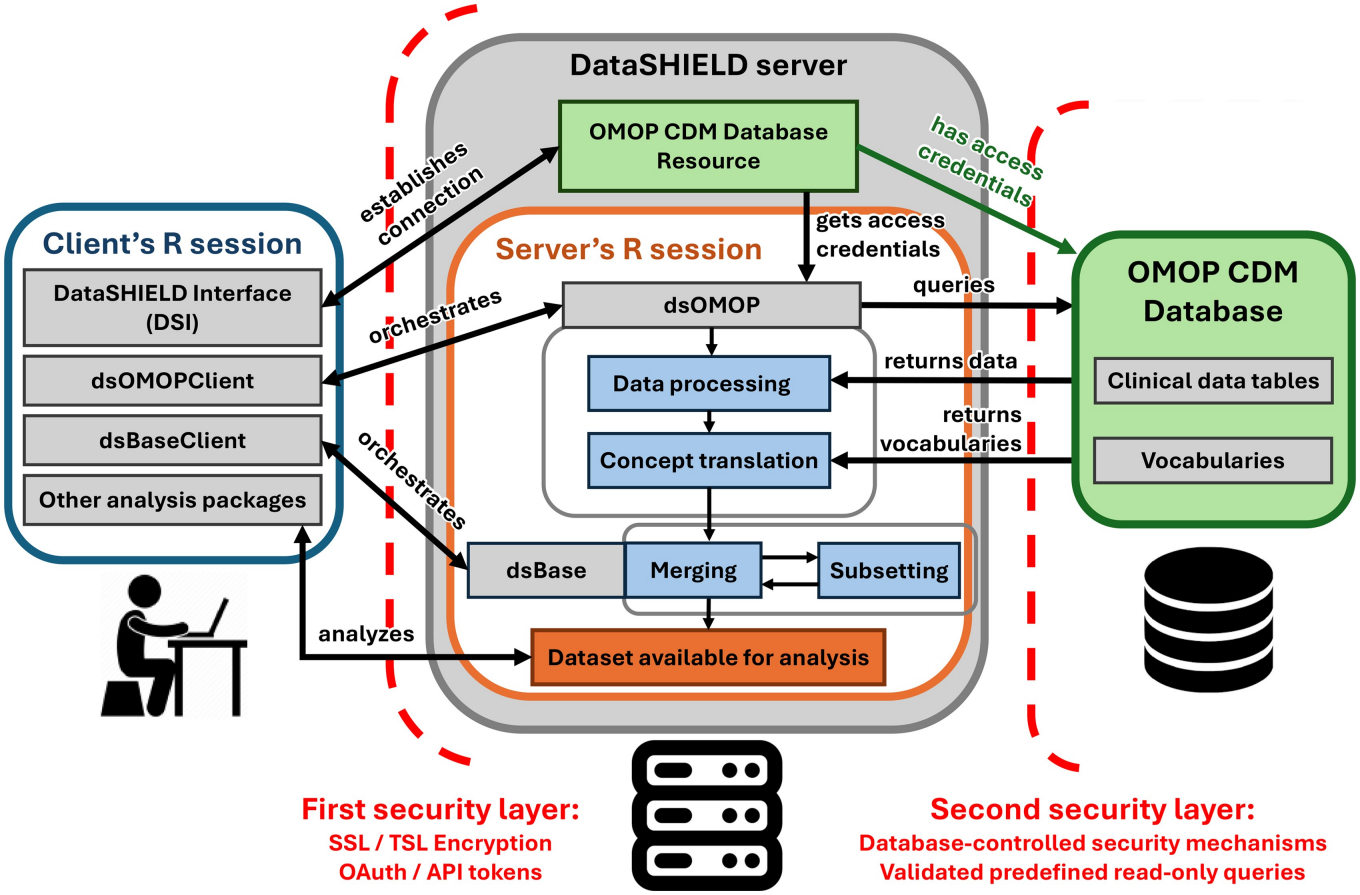
*dsOMOP*’s functioning architecture. It illustrates the processes and interactions between each component within the system.

The *dsOMOP* framework is divided into two R-based software components; namely, *dsOMOP* (server-side) and *dsOMOPClient* (client-side), which work together to facilitate the framework’s functionality. The primary objective of these packages is to enable researchers to remotely construct a dataset tailored to their specific research needs through the *dsOMOPClient* client-side interface on their local machine, while the actual dataset assembly occurs in an isolated R session on the *DataSHIELD* server under the control of the server-side package *dsOMOP* (see Fig. 1). This approach ensures that all subsequent data analyses take place on the server and that only aggregated (non-disclosive) results are returned to the client, thus maintaining the safeguards of *DataSHIELD*’s federated design.

#### 2.1.1 Server-side package

The *dsOMOP* package is installed and operates on the *DataSHIELD* server, which is typically deployed within the infrastructure of a participating institution in the research network. These servers are managed by the data-owning sites themselves, ensuring that the data remains under their control and governance. They also determine which server-side packages are installed, thereby selecting the functionalities available for the analysis of their data.

This package is responsible for interacting with OMOP CDM-compliant databases to fetch specified data, processing it according to the user’s parameters, and securely constructing the desired datasets and loading them into the R session on the server. All these operations are executed based on highly customizable function calls from *dsOMOPClient*, which are orchestrated remotely by researchers from their local machines.

The communication between the server and the database adheres to the database’s intrinsic security configurations, including authentication protocols, access controls, and encryption mechanisms. All queries executed by the *dsOMOP* package are limited to those defined within the package’s programming and have been validated to ensure they are safe and secure.

#### 2.1.2 Client-side package

The *dsOMOPClient* package is installed on the researcher’s local system and provides a suite of methods to define and coordinate server-side data retrieval operations and prepare datasets for subsequent analysis within the *DataSHIELD* environment. It allows users to explore the database schema and instruct the server to assign filtered OMOP CDM tables to internal R session symbols while automatically applying relevant filters. The process is highly customizable, with parameters that enable users to request data retrieval and processing operations on the server that align with their target dataset requirements. The client-side package ensures a user-friendly interface for specifying data needs and requirements and facilitates efficient interactions with the server-side functionalities.

Importantly, *dsOMOPClient*’s responsibilities are limited to initiating and managing these data extraction and cohort construction steps; it does not itself perform any statistical aggregation or analysis on the retrieved information. Once the requested data resides in the server’s R session, subsequent summarization or analytical computations are orchestrated by *DataSHIELD*’s analytical client packages (for example, by invoking server-side *dsBase* functions via the *dsBaseClient* package).

When establishing communication with the *DataSHIELD* server, *dsOMOPClient* inherits the robust security mechanisms of *DataSHIELD*’s architecture. These include encrypted *SSL*/*TLS* protocols to ensure secure data transmission, and authentication measures such as *API* tokens or *OAuth*, which restrict access to authorized users.

### 2.2 Server–database interaction

#### 2.2.1 Resource creation and management

*DataSHIELD*’*s* architecture was significantly expanded beyond its traditional format of handling tables within the server’s storage following the introduction of the *resourcer* package (Marcon *et al.* 2021). This extension enables *DataSHIELD* to interact with external data sources, including remote databases, thereby enhancing its capability to perform federated analyses on a broader variety of datasets.

The *resourcer* package introduces the creation and management of resources, allowing package developers to incorporate support for new data formats and types within the *DataSHIELD* environment. This includes defining connection parameters for remote databases, which are then treated as external components and can be read by dedicated server-side packages. The *dsOMOP* package uses this expanded architecture to create resources specifically dedicated to OMOP CDM databases.

#### 2.2.2 Data fetching optimization

*dsOMOP* performs data fetching operations by sending optimized SQL queries from the server’s R session to the external OMOP CDM databases. These queries are designed to retrieve the specified data as requested by the researcher, applying selection filters directly at the database level to optimize retrieval times. By filtering data during query execution, *dsOMOP* significantly reduces the computational load on the R session and ensures that only the necessary data is transferred to the server’s session, enhancing performance and minimizing the handling of large volumes of data, which are typical for OMOP CDM databases.

The fetched data retains a filtered view based on patient, concept (a standardized unit of meaning for clinical entities such as conditions, drugs, or procedures), and column selection from the original OMOP CDM tables, ensuring the preservation of structure and relationships for subsequent processing steps. These steps enable the transformation of the data into an abstracted representation aligned with the specific research objectives.

### 2.3 Data disclosure control

*DataSHIELD* incorporates various data disclosure control settings that can be configured by data owners from the participating institutions. One of their responsibilities in the maintenance and setup of a *DataSHIELD* server is to configure the level of strictness with which *DataSHIELD* enforces its data disclosure control measures. An example of these settings is the nfilter.subset, which defines the minimum non-zero count of observational units (typically individuals) in a subset. This filter serves as a safeguard to mitigate disclosure risk by preventing outputs containing potentially identifiable individual-level information.

**Figure 2.**
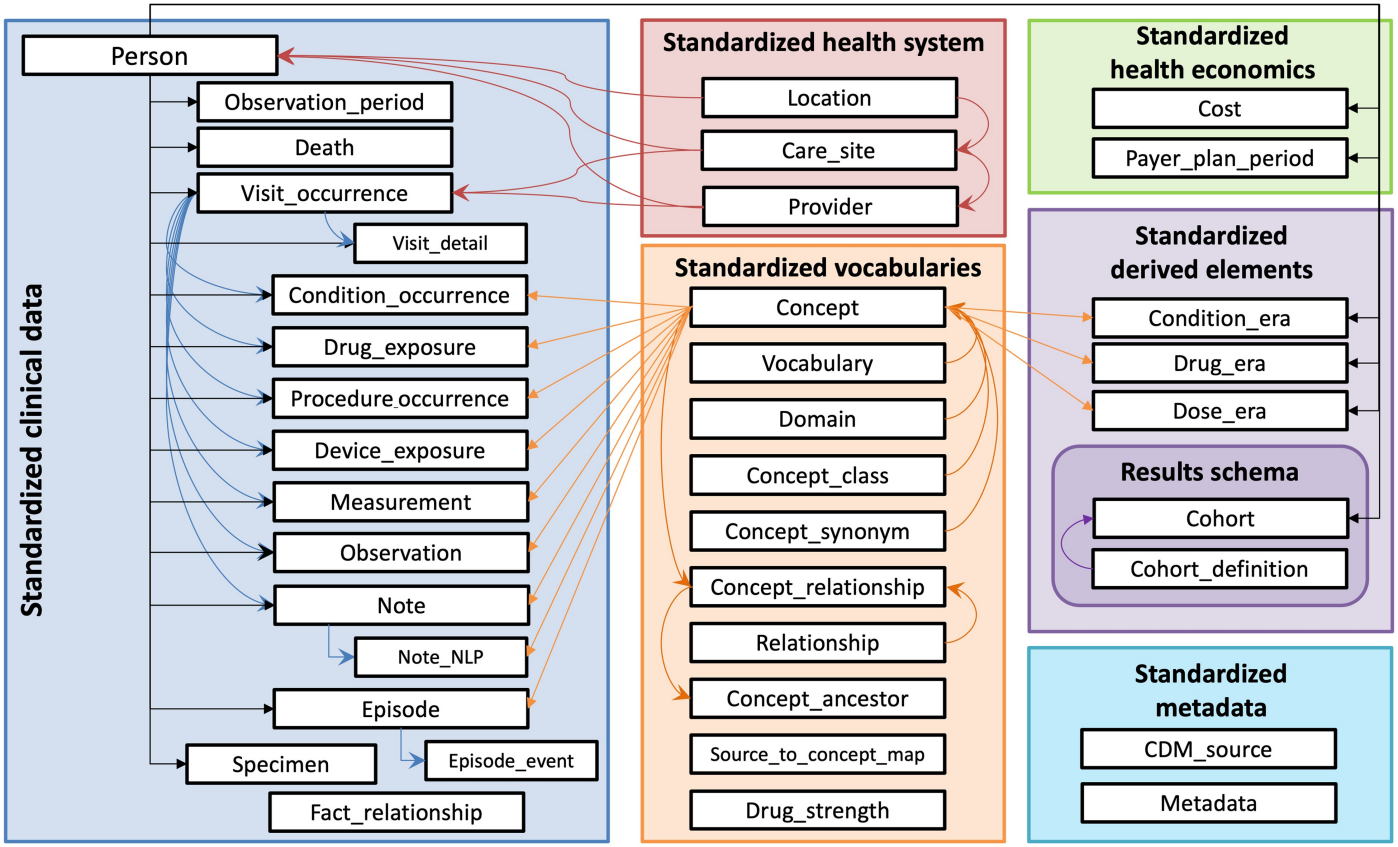
OMOP CDM v5.4 Schema. It illustrates the structure and relationships of tables and fields within the version 5.4 of the OMOP Common Data Model (CDM). *Reproduced from OHDSI’s OMOP Common Data Model Wiki (CC BY-SA 4.0)*.

Traditionally, datasets used by *DataSHIELD* have a one-to-one correspondence between records and individuals, such as a wide-format registry table where each row represents a single individual, and columns correspond to measured data elements. However, OMOP CDM databases contain longitudinal data in a relational structure made up of multiple records per individual distributed across different tables. More specifically, data in OMOP CDM format is organized into domains (e.g. measurement, condition), each of which has its own table that contains domain-specific concepts (see Fig. 2).

A clinical condition in OMOP CDM, such as a diagnosis of asthma, is thus represented by a unique row in the condition-specific table that references identifiers in other tables describing (i) the individual who was diagnosed, (ii) the standard concept identifier for asthma, (iii) the date on which the diagnosis was recorded, (iv) the encounter in which the diagnosis was made, and (v) other information related to the diagnostic procedure and health system. To accommodate this relational complexity, *dsOMOP* adapts the nfilter.subset check to ensure compliance with *DataSHIELD*’s disclosure control standards: during the information processing phase, *dsOMOP* considers the nfilter.subset and identifies the number of unique individuals represented in any dataset extracted from the database. If the number of individuals in the subset falls below the threshold set by nfilter.subset, the data is automatically identified as disclosive and excluded by *dsOMOP* during dataset construction and from the data catalog during exploration operations. This feature extends the in-built *DataSHIELD*’s disclosure control mechanisms that ensure that sub-setting thresholds are applied not only to rows representing individual patients but also to all data related to those patients across multiple tables in the OMOP CDM.

Additionally, all data transformation functions executed internally from the server-side package directly call functions from *DataSHIELD*’s *dsBase* package. These functions have been audited by the *DataSHIELD* community and incorporate disclosure checks. By using these functions directly from the *dsBase* package, any updates or additional measures incorporated into the original package will take effect immediately in *dsOMOP* when the data owner updates the *dsBase* package on the server.

### 2.4 Data processing operations

The *dsOMOP* package performs a series of data transformation operations on the datasets retrieved directly from the database. These processes are designed to manipulate the datasets into a user-friendly and easily interpretable structure that expedites data analyses and investigations.

#### 2.4.1 Concept columns formatting

*dsOMOP* automatically reformats tables containing multiple events linked to a single concept into a wide format table during processing on the server. This transformation, carried out after retrieving individual OMOP CDM tables from the database, generates a separate column for each attribute related to each concept type present in the table, aligning with the traditional *DataSHIELD* approach of a one-record-per-patient view.

To address the computational challenges inherent in wide-format data, such as increased memory usage and slower computation for complex analyses, *dsOMOP* emphasizes the exclusion of unnecessary columns and records during data fetching and dataset construction, reducing the computational load while tailoring an abstracted view for specific use cases.

#### 2.4.2 Relational column detection and CDM version-agnostic approach

The *dsOMOP* package includes an automated method for detecting relational columns within tables using pattern recognition to identify and adapt them. When a table contains multiple records linked to the same entity, the method restructures the table to prepare it for merging with other extracted tables, preserving foreign key columns to maintain relationships and reshape the dataset according to the structural requirements of downstream analysis or merging with other tables.

This functionality is specifically designed to address the structural variations present across different versions of the OMOP CDM. Such variations often include modifications in table distribution, naming, or domain organization. However, *dsOMOP* is purposely designed to be CDM version-agnostic, meaning it processes data in a way that does not depend on a single fixed schema or any specific iteration of the OMOP CDM. Rather than referencing hard-coded table layouts, *dsOMOP* scans the data model at runtime to dynamically detect how domain tables link to one another through their foreign keys.

In many practical implementations, the *Person* table often provides a common anchor for referencing patient-level information. Nonetheless, *dsOMOP* does not require using any specific table as a mandatory entry point; it simply identifies the relational patterns that connect whichever tables the user wishes to analyze. Even when a piece of information is linked across multiple intermediary references from the central *Person* record, *dsOMOP*’s dynamic table mapping will locate and incorporate it without any version-specific adaptation.

By reconstructing these references dynamically, *dsOMOP* can automatically adapt to typical modifications introduced in different CDM versions—such as renaming or subdividing tables—without needing version-specific modifications to the software. This approach allows researchers to build datasets in a uniform manner across multiple OMOP CDM variants, ensuring that *dsOMOP* remains applicable even as the CDM evolves over time.

#### 2.4.3 Concept translation

Categorical information in OMOP CDM tables is represented using concept IDs (e.g. a measurement of SARS-COV2 antigen might have an associated concept value of *9191* meaning *Positive*), which refer to predefined concepts in the database vocabularies. *dsOMOP* identifies all concept IDs present in the database and automatically translates them to their textual names as defined in the database vocabulary. This translation facilitates a more intelligible and user-friendly format for researchers.

To address potential discrepancies between database vocabulary versions across sites within a network, *dsOMOP* relies on each site’s locally defined vocabulary version by retrieving the concepts directly from the vocabulary data tables. This ensures that translations are performed consistently with the specific database version in use at each site. It is recommended that participating sites in a multicenter network harmonize their vocabulary versions during the data harmonization process to minimize inconsistencies in cross-site research. Additionally, a large proportion of concepts in OMOP CDM databases are standardized within widely used public vocabularies, such as SNOMED, LOINC, and RxNorm. As a result, these concepts are typically consistent across sites, which supports their use in accurate multi-site research.

#### 2.4.4 Longitudinal data processing

OMOP CDM supports longitudinal data, where multiple records of the same type may exist for the same entity over time (e.g. multiple hemoglobin measurements over the course of a year). *dsOMOP* offers flexibility in how these repeated measurements are handled. By default, such longitudinal data remain in a long format as separate records for each patient. This approach preserves all individual observations and allows researchers to apply *DataSHIELD*’s built-in non-disclosive aggregation functions (for example, computing a mean or other summary across a patient’s repeated measurements). Alternatively, *dsOMOP* can restructure the data into a wide format, producing one record per patient with multiple time-indexed columns for the values of that attribute. The choice of long versus wide representation is left to the researcher and depends on the specific analytical needs.

However, a challenge in wide-format longitudinal data is that different individuals may have observations recorded at different time points. To address this, *dsOMOP* introduces an optional parameter, completeTimePoints, to standardize time indices across individuals. When completeTimePoints is enabled, the data processing function identifies the full set of time points observed in the dataset for the given attribute (across all participants) and inserts missing entries for any participant who lacks a record at a particular time. This ensures that each participant’s data includes the same set of time points (with NA placeholders where data are absent), enabling a consistent structure for longitudinal analysis across all individuals (for example, if one patient has a measurement at a time point that another patient does not, the latter will have an NA entry for that time point in the wide-format output). Standardizing the time axis in this way facilitates straightforward comparisons over time and simplifies data preparation operations and downstream analyses on the combined dataset.

### 2.5 Incorporation of data into *DataSHIELD*’s workflow

*dsOMOP* automatically makes the retrieved data available within the *DataSHIELD* workflow, ensuring that all future operations adhere to *DataSHIELD*’s disclosure control technical measures. This process begins as soon as the individual tables have been extracted from the OMOP CDM databases and processed, with the resulting tables being incorporated into the server’s R session. Once these tables are available in the server session, any further analysis or aggregation is performed using *DataSHIELD*’s analytical packages, with *dsOMOP* and *dsOMOPClient* no longer involved beyond the dataset construction stage. This separation of roles allows *dsOMOP* to focus on the retrieval and construction of the standardized dataset while leveraging *DataSHIELD*’s established analysis framework for all statistical operations.

The extracted tables often need to be unified through merging operations to construct the desired final dataset. This process is achieved using commands from the *dsBaseClien*t package, which calls the methods of the server-side package *dsBase*. In combination with merging operations, users can also perform sub-setting operations to shape the dataset and guide its creation process.

The package optimizes data retrieval and processing times by including a parameter in its data retrieval function that allows for the specification of an existing table in the server’s R session. This function will then extract the patient identifiers from that table (without making them specifically accessible to the user) and force subsequent queries to select only data from those patients in other tables. This allows researchers to create specific cohort definitions tailored to their study needs and avoid data redundancy.

### 2.6 Support for community extensions

#### 2.6.1 Extensibility of the client-side package

The design of *dsOMOPClient* is fundamentally focused on serving as an interactor with *dsOMOP*, which in turn interacts with the external databases. This means that *dsOMOPClient* offers essential functionalities for database interaction but does not automatically perform all the subsequent unification steps needed to construct the final dataset. This approach provides the necessary flexibility for defining cohorts and creating datasets to accommodate any possible use case. Consequently, there are certain operations that users need to perform once the dataset has been incorporated into *DataSHIELD*’s workflow.

To support wide-ranging use cases, *dsOMOPClient* can incorporate third-party developers’ packages and routines. These extensions enhance the usability and automation of existing functionalities by leveraging *dsOMOP*’s capabilities in combination with *DataSHIELD*’s base operations. Despite this flexibility, all actions performed on the data are executed through the server-side disclosure controls provided by *dsOMOP* and core *DataSHIELD* operations. Consequently, third-party packages developed in alignment with the methods provided by *dsOMOP* are expected to inherently comply with *DataSHIELD*’s fundamental disclosure control standards.

To demonstrate the vast potential of this design, we developed a supplementary R package named *dsOMOPHelper*, designed to showcase and provide a functional set of tools that cover the complete automation of dataset creation for most common use cases of *dsOMOPClien*t, albeit with less flexibility in the process. *dsOMOPHelper* leverages the combination of commands from both *dsOMOPClient* and *dsBaseClient* to enable researchers to quickly construct highly customizable datasets within a few commands, ready to be used in the *DataSHIELD* environment.

While the combination of *dsOMOPHelper* for general cases and *dsOMOPClient* for edge cases or those requiring more flexibility may suffice for working with *dsOMOP*, our goal is to create a collaborative ecosystem where developers can contribute by building additional packages based on *dsOMOPClient* that address particular use cases for various studies. Such community-driven development would expand the capabilities for working with the combined capabilities of *DataSHIELD* and OMOP CDM and promote a cooperative environment between their respective communities.

#### 2.6.2 Extensibility of the server-side package

*dsOMOP* works in conjunction with the *resourcer* package, which manages the connection and interaction with databases from various database management systems (DBMS). It is possible to create extensions of *resourcer* that register new types of data sources (e.g. the *s3.resourcer* package, which enables access to files stored in the *AWS S3* system). This extensibility can be used to enable dsOMOP to interact with a wider range of database systems.

However, the *resourcer* package does not handle the adaptability required for database schema selection queries, which often vary in syntax depending on the DBMS. Since these queries are essential for *dsOMOP*’s functionality, the package includes features for registering new schema interaction query syntax to accommodate these differences. This registration, in combination with *resourcer* package extensions, facilitates the creation of *dsOMOP* extensions that expand its interoperability to other DBMS.

An example of this is the *dsOMOP*.*oracle* package, which extends dsOMOP’s operability to Oracle databases. This package works on top of oracle.resourcer, an extension developed specifically to enable resourcer to interact with Oracle-based systems. Together, *dsOMOP.oracle* and oracle.resourcer were developed as a pilot showcasing *dsOMOP’s* extensibility for DBMS interoperability. It serves as an educational example of how to leverage *dsOMOP* and the *resourcer* package to create operability extensions, while also addressing the needs of institutions that work with Oracle databases.

### 2.7 Validation methodology

#### 2.7.1 Reproducibility of studies from the scientific literature

In this work, we demonstrate the functionality and utility of *dsOMOP* in a simulated distributed multicenter study environment, with the objective of replicating analyses from published scientific studies. By conducting these analyses across multiple databases, we seek to verify that the results are consistent with findings reported in the literature.

To replicate real-world collaborative research, we established an infrastructure where data from multiple institutions remains securely stored in their original databases while being accessible for federated analyses via *DataSHIELD*. Each database is connected to a *DataSHIELD* server through a resource. All communication and access occur exclusively within the *DataSHIELD* framework, ensuring that interactions with the database are confined to the server of the institution that owns the data, maintaining a strict separation of access and safeguarding the integrity and confidentiality of institutional data.

#### 2.7.2 Data overview

For this reproducibility analysis, we utilized the Tufts Synthetic Dataset, which consists of fully synthetic electronic health record (EHR) data representing 567 000 synthetic patients. This dataset was generated in 2021 through a collaboration between Syntegra, Inc. and Tufts Medical Center using a deep learning transformer model. The model was trained on real-world EHR data from the Tufts Research Data Warehouse (TRDW), which had already been transformed into OMOP CDM format from other underlying data structures. It includes longitudinal clinical data from patients who received care across Tufts Medicine’s three hospitals, 40-practice physician network, and home health care organization.

The Tufts Synthetic Dataset contains clinical information such as patient visits, conditions, medications, laboratory measurements, procedures, observations, and device exposures, all structured according to the OMOP CDM version 5.3. The large volume of patient data, along with the realistic nature of the synthetic dataset, makes it ideal for testing the functionality of *dsOMOP* by exploring significant associations and patterns within the data, much like those expected in real-world clinical datasets.

## 3 Results

We aimed at identifying key predictors for chronic obstructive pulmonary disease (COPD) based on their relevance in the literature while considering the availability and representation of those variables in our dataset. To this end, we selected as predictors a set of variables which has shown well-established associations with COPD described in the review by Holtjer *et al.* (2023):

- Tobacco use
- Vitamin D deficiency
- History of asthma
- History of rheumatoid arthritis

**Figure 3.**
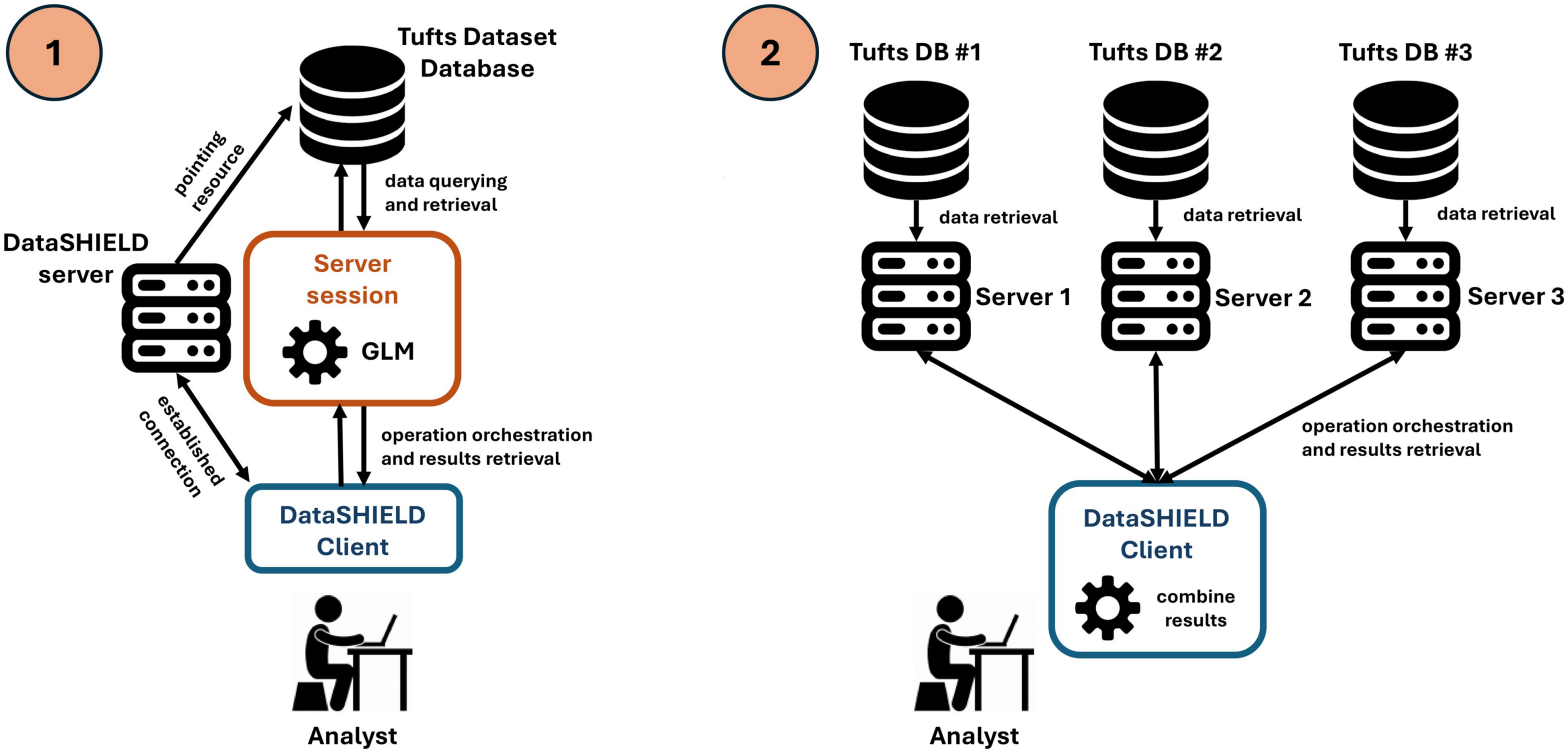
Single versus distributed data environments. In the distributed setup (right), processes are simplified for visualization, but the same operations—data retrieval, analysis, and result combination—are conducted simultaneously across multiple servers before being aggregated on the client-side.

To evaluate the relationship between the potential predictors and the presence of COPD, we performed a generalized linear model (GLM) analysis within the *DataSHIELD* framework. This model was conducted using a binomial family and a logit link function, allowing us to assess the odds ratios (OR) for each predictor. To validate the federated approach, we compared the results obtained from executing the GLM in two distinct scenarios. These scenarios are illustrated in Fig. 3:

1. The entire Tufts Synthetic Dataset was processed as a single dataset within a single *Opal* server.
2. The Tufts Synthetic Dataset was divided into three parts, each hosted on a separate *Opal* server, with connections established via the *DataSHIELD* client. Each server connected to a resource pointing to its respective database, containing a portion of the patients from the dataset.

In both scenarios, the GLM was executed, and the results from the federated analysis were found to be identical to those obtained in the pooled analysis, demonstrating *dsOMOP*’s capabilities to reliably reproduce statistical results of distributed analyses in federated environments. Results are depicted in Table 1. The code detailing how these estimates are computed within the *DataSHIELD* environment is available at: https://isglobal-brge.github.io/dsOMOPHelper/articles/copd.html.

**Table 1.** Odds ratios (OR), 95% confidence interval (CI) and *P*-values for predictors of COPD, obtained from both the single-site (pooled) and distributed (federated) GLM analyses.^a^

Variable	OR (CI 95%)	*P*-value
Tobacco use	6.43 (3.98–10.37)	2.50 × 10⁻¹⁴
Vitamin D deficiency	6.14 (3.36–11.23)	3.81 × 10⁻⁹
History of asthma	20.02 (15.99–25.08)	4.46 × 10⁻¹⁵⁰
History of rheumatoid arthritis	8.22 (3.76–18.00)	1.37 × 10⁻⁷

a Since both analyses produced identical values, a single set of results is presented.

## 4 Discussion

### 4.1 Validation of dsOMOP for federated clinical research using real-world data

The generalized linear-model results confirm that each selected predictor—tobacco use, vitamin D deficiency, history of asthma and rheumatoid arthritis—remains significantly associated with COPD (all ORs > 1, *P* < 0.05). Obtaining identical estimates in the pooled and fully distributed analyses demonstrates that *dsOMOP* can reproduce literature-known associations while respecting *DataSHIELD*’s disclosure controls. In practice, this means researchers can analyse OMOP CDM data across multiple centres without moving patient-level records, yet still achieve statistically robust, fully comparable outputs.

### 4.2 Software availability

All the software developed for this project, including *dsOMOP*, *dsOMOPClient*, *dsOMOPHelper*, and *dsOMOP.oracle*, is available for installation from the GitHub repositories of the Bioinformatics Research Group in Epidemiology (BRGE) at the Barcelona Institute for Global Health (https://github.com/isglobal-brge), and a permanent archival snapshot of the exact code used in this manuscript is deposited at *Figshare* (https://doi.org/10.6084/m9.figshare.28607186). To help researchers familiarize themselves with these tools, the materials include complementary vignettes with hands-on examples. These educational resources are available on the corresponding websites of the repositories (https://isglobal-brge.github.io/dsOMOPClient and https://isglobal-brge.github.io/dsOMOPHelper).

### 4.3 Software demonstration

A version of *dsOMOP* is available for public access and testing on the OBiBa Opal demonstration server (https://opal-demo.obiba.org), along with a testing database which is freely available and does not require to sign any data transfer agreement (MIMIC IV in the OMOP CDM format).

The MIMIC IV (Medical Information Mart for Intensive Care) database is a freely available critical care database that includes de-identified health data associated with approximately 40 000 critical care patients. The data encompasses vital signs, laboratory results, medications, and more, collected from the intensive care units (ICUs) of the Beth Israel Deaconess Medical Center between 2008 and 2019 (Johnson et al., 2023).

For the demonstration, we uploaded the data of 100 patients from the standardized version of the MIMIC IV database in the OMOP Common Data Model (CDM), as provided by the work of Kallfelz *et al.* (2021). Instructions for connecting and using *dsOMOP* on the demonstration server with the MIMIC IV database, along with general practical examples, can be found in the usage vignettes on the websites of *dsOMOPClient* and *dsOMOPHelper.*

## 5 Conclusion

*dsOMOP* represents a significant advancement in collaborative clinical research by bridging the gap between data standardization and disclosure-controlled federated data analysis. By enabling the incorporation of OMOP CDM data within the *DataSHIELD* framework, *dsOMOP* enables research institutions to analyze standardized clinical data across multiple centers without transferring individual-level patient data, thus significantly reducing disclosure risks. Our illustrative data analysis using Tufts Synthetic Dataset validates the technical and analytical capabilities of *dsOMOP* in the replication of complex clinical studies, highlighting its effectiveness and potential to facilitate federated clinical research in distributed data environments while working with large-scale, standardized clinical data.

These interoperability capabilities not only simplify multicenter data analyses but also aligns with evolving data protection regulations, emphasizing controlled data disclosure practices in large-scale clinical research projects. The availability of *dsOMOP* as an open-source tool further encourages widespread adoption and customization, fostering collaborative research and supporting the scientific community’s need for secure, standardized data analysis in a federated framework.

## Data Availability

This study analyzed only existing datasets; no new patient-level data were generated. The Tufts Synthetic Dataset was provided by Tufts Medical Center and Syntegra, Inc. under license; access is restricted and may be granted to qualified researchers upon request to the corresponding author, subject to a separate data-use agreement and prior written approval from Tufts Medical Center. The publicly available MIMIC-IV demo dataset, converted to the OMOP Common Data Model (version 0.9), can be accessed in the PhysioNet repository at https://doi.org/10.13026/p1f5-7x35.
